# Proton-conductive coordination polymer glass for solid-state anhydrous proton batteries†

**DOI:** 10.1039/d1sc00392e

**Published:** 2021-03-12

**Authors:** Nattapol Ma, Soracha Kosasang, Atsushi Yoshida, Satoshi Horike

**Affiliations:** Department of Synthetic Chemistry and Biological Chemistry, Graduate School of Engineering, Kyoto University Katsura, Nishikyo-ku Kyoto 615-8510 Japan horike@icems.kyoto-u.ac.jp; Department of Chemical and Biomolecular Engineering, School of Energy Science and Engineering, Vidyasirimedhi Institute of Science and Technology Rayong 21210 Thailand; AIST-Kyoto University Chemical Energy Materials Open Innovation Laboratory (ChEM-OIL), National Institute of Advanced Industrial Science and Technology (AIST) Yoshida-Honmachi, Sakyo-ku Kyoto 606-8501 Japan; Institute for Integrated Cell-Material Sciences, Institute for Advanced Study, Kyoto University Yoshida-Honmachi, Sakyo-ku Kyoto 606-8501 Japan; Department of Materials Science and Engineering, School of Molecular Science and Engineering, Vidyasirimedhi Institute of Science and Technology Rayong 21210 Thailand

## Abstract

Designing solid-state electrolytes for proton batteries at moderate temperatures is challenging as most solid-state proton conductors suffer from poor moldability and thermal stability. Crystal–glass transformation of coordination polymers (CPs) and metal–organic frameworks (MOFs) *via* melt-quenching offers diverse accessibility to unique properties as well as processing abilities. Here, we synthesized a glassy-state CP, [Zn_3_(H_2_PO_4_)_6_(H_2_O)_3_](1,2,3-benzotriazole), that exhibited a low melting temperature (114 °C) and a high anhydrous single-ion proton conductivity (8.0 × 10^−3^ S cm^−1^ at 120 °C). Converting crystalline CPs to their glassy-state counterparts *via* melt-quenching not only initiated an isotropic disordered domain that enhanced H^+^ dynamics, but also generated an immersive interface that was beneficial for solid electrolyte applications. Finally, we demonstrated the first example of a rechargeable all-solid-state H^+^ battery utilizing the new glassy-state CP, which exhibited a wide operating-temperature range of 25 to 110 °C.

## Abstract 2

Melt-quenched coordination polymer glass shows exclusive H^+^ conductivity (8.0 × 10^−3^ S cm^−1^ at 120 °C, anhydrous) and optimal mechanical properties (42.8 Pa s at 120 °C), enables the operation of an all-solid-state proton battery from RT to 110 °C.

## Graphical abstract

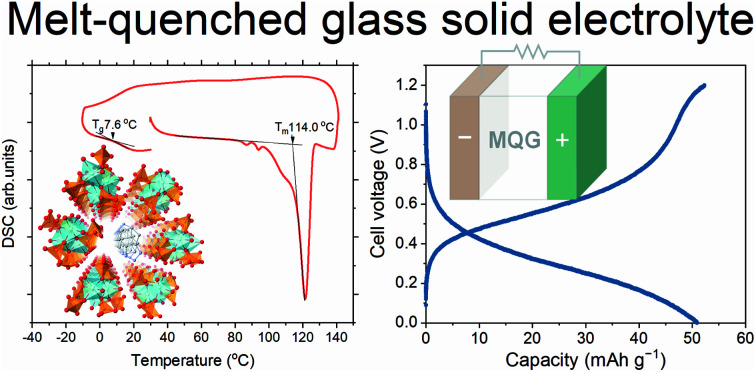

## Introduction

The proton (H^+^) has a diameter of 0.84 fm and is easily localized in the solid state.^1^ Fast-moving protons in solids are difficult to achieve, whereas solid-state H^+^ conductors are widely used in various electrochemical applications, including fuel cells, electrochemical catalysis, and sensors.^2^ Proton batteries are a new class of secondary batteries employing protons instead of metal ions as charge carriers.^3,4^ They consist of faradaic electrodes and acidic electrolytes. Since the H^+^ charge radius is significantly smaller than that of other ions, faster ion migration and negligible volume changes upon H^+^ insertion/desertion are expected. Additionally, replacing high-cost Li^+^ with cheaper and more abundant H^+^ provides a promising platform for environmentally benign and intrinsically safe energy storage.^5–7^ Redox-active organic molecules, such as quinone-functionalized conductive polymers,^4,8^ and metal oxides, including MoO_3_, WO_3_, and H_*x*_IrO_4_, are available as H^+^ electrodes.^9–11^ Though proton batteries show a smaller specific capacity with a limited number of applications, as compared to their metallic counterparts, diffusion-free charge transport *via* the Grotthuss mechanism in a defective, Prussian blue analog establishes a high-rate capability (380 A g^−1^) and extends cycling stability to over 0.5 million charge–discharge cycles, which is a unique advantage of aqueous proton batteries.^6,12,13^ In spite of various choices of electrodes, electrolytes are mostly limited to aqueous H_2_SO_4_ or H_3_PO_4_, which dictates the operating-temperature window and selection of usable electrodes.^8,13–15^

Safely extending the operating-temperature window to ∼100 °C is essential for H^+^ batteries to tolerate internal/external heat generation so that they can be used in various high-temperature applications, such as rescue/inspection robots, space exploration, and measure-while-drilling (MWD) equipment in the oil and gas industries.^16^ As employing a conventional aqueous electrolyte is not possible at these high temperatures, solid-state H^+^ batteries with anhydrous solid electrolytes would be more suitable. There are no reports of solid-state H^+^ batteries working near or above 100 °C due to the difficulties in achieving high anhydrous H^+^ conductivity, high-temperature stability, and moldability required for H^+^ conductors.^17^ Apart from achieving a high H^+^ conductivity value (near 10^−2^ S cm^−1^), high thermal/chemical stability, processing ability, and ion selectivity are also needed to expand the practicality of solid-state electrolytes. Single-ion conductivity in solid-state electrolytes is a core factor that promotes charge-transport efficiency and prevents anion polarization.^18,19^ Discontinuities along the electrode–electrolyte interfaces and grain boundaries are primary bottlenecks for efficient utilization of solid electrolytes.^17,20,21^ H^+^ conductivity at the grain boundary of most crystalline compounds requires a higher migration activation energy than that required by H^+^ conductivity through the bulk crystal (grain boundaries contribute up to 40–50% of the overall resistance for Li^+^ conductors).^22–25^

Coordination polymers (CPs) and metal–organic frameworks (MOFs) exhibiting high H^+^ conductivity over a wide temperature regime (∼200 °C) represent a new class of solid-state H^+^ conductors.^26–29^ Despite their remarkable H^+^ conductivity, their crystalline nature hinders their processing ability, thus limiting their practicality.^30^ The glassy state of CP/MOFs is a strong platform to tackle these issues, and there have been increasing numbers of glassy-state CPs recently made from crystalline-state CPs.^31–34^ Some of these glassy-state CPs show anhydrous H^+^ conductivity superior to that of their crystalline counterparts by several orders of magnitude.^35,36^ Moreover, the vitrifying/melting behavior provides these CPs with processing capabilities and forms a grain-boundary free monolith and a flawless heterogeneous interface.^31–34,37–41^

To address this issue, we have developed a new H^+^-conductive CP glass suitable for high-temperature anhydrous solid-state H^+^ batteries. By optimizing the p*K*_a_ value of the component with 1,2,3-benzotriazole (BTA, p*K*_a_ 1.6) and the extended hydrogen-bonding network in Zn^2+^-based CPs, the material demonstrated high anhydrous H^+^ conductivity (8.0 × 10^−3^ S cm^−1^ at 120 °C), relatively low melting point (114 °C), and mechanical softness (42.8 Pa s at 120 °C), which are suitable for electrolytes. The structure and properties were characterized by single-crystal X-ray diffraction (SC-XRD), thermal gravimetric analysis (TGA), differential scanning calorimetry (DSC), dynamic mechanical analysis (DMA), impedance spectroscopy, electromotive force measurements, and solid-state NMR. We also demonstrated a full-cell evaluation of the anhydrous solid-state H^+^ batteries at 25, 100, and 110 °C.

## Results and discussion

### Crystal structure of [Zn_3_(H_2_PO_4_)_6_(H_2_O)_3_](BTA) (**1a**)

**Fig. 1:**
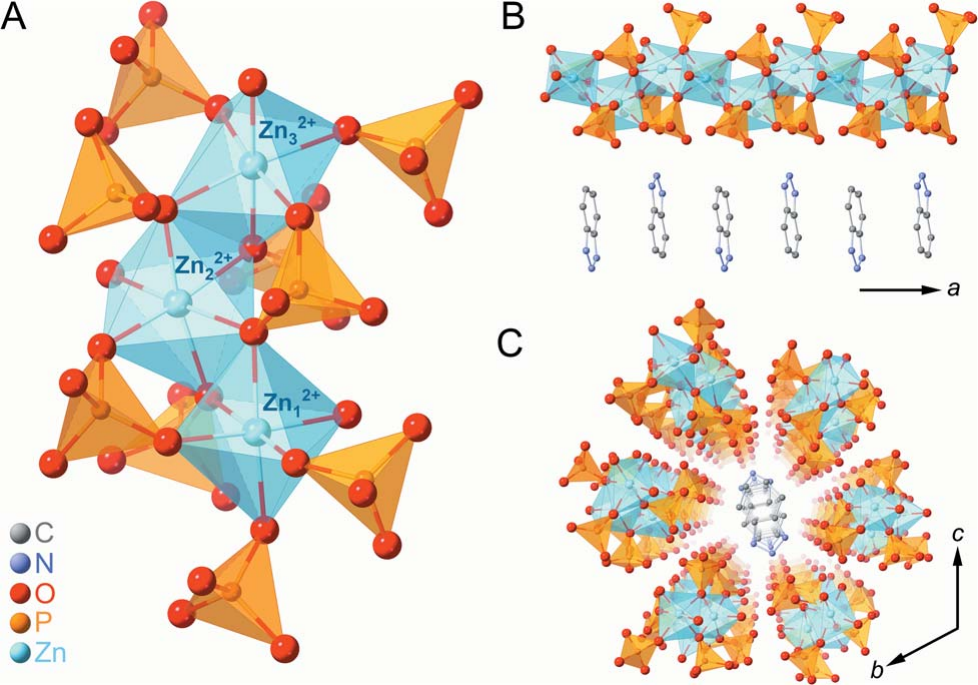
(A) Local coordination geometry in **1a**. (B) Crystal structure of the one-dimensional (1D) chain along the *a*-axis. (C) Packing structure of **1a** on the *bc*-plane. Zn, P, O, C, and N atoms are represented by light blue, orange, red, grey, and blue spheres, respectively. H atoms are omitted for clarity.

Zinc oxide, phosphoric acid, and BTA were subjected to mechanical milling to form the CP (**1a**) as a white crystalline powder. SC-XRD analysis of **1a** provided its chemical formula, [Zn_3_(H_2_PO_4_)_6_(H_2_O)_3_](BTA), and it was found to exist as a one-dimensional (1D) chain along the *a*-axis (Fig. 1). Three crystallographically independent octahedral Zn^2+^ ions were identified, each with six bridging H_2_PO_4_^−^ anions and one water molecule coordinated to them (Fig. 1A). BTA was stacked in a 1D fashion along the *a*-axis and surrounded by six chains of ZnO_6_ octahedra, which orderly arranged in the *bc* plane due to hydrogen-bonding interactions (Fig. 1B and C). Furthermore, **1a** is an isostructure of previously reported [Zn_3_(H_2_PO_4_)_6_(H_2_O)_3_](benzimidazole),^38^ and it is expected that the dynamics of the phosphates bridging the Zn^2+^ ions (through a single bridging oxygen atom (μ_2_)) and the non-coordinating BTA could facilitate an anhydrous H^+^ migration.^2,38,42,43^

The gram-scale synthesis of **1a** was feasible *via* mechanical milling for 1 h followed by vacuum drying for 3.5 h to remove excess water molecules. Powder X-ray diffraction (PXRD) of **1a** (Fig. S1†) demonstrated a pattern identical to the simulated SC-XRD pattern. The absence of residual free phosphoric acid in **1a** was confirmed using both inductively coupled plasma emission spectroscopy (ICP-ES) and ^31^P magic-angle spinning (MAS) solid-state NMR (Fig. S2†).^44^ A P to Zn ratio (1 : 1.97) slightly lower than the theoretical ratio (1 : 2) suggested the presence of a small amount of structural defects. All peaks in ^31^P NMR were located in the range of orthophosphate, suggesting that no condensation occurs during the mechanical synthesis.^45–47^ TGA of **1a** showed a gradual weight loss due to the release of coordinated water at 100 °C (Fig. S4†). The total weight loss of dehydrated **1a** is equivalent to the release of three coordinated water molecules (5.7 wt%). This dehydrated state is henceforth denoted as **1**. A reversible structural change between **1a** and **1** upon water adsorption and desorption was observed by PXRD (Fig. S5†).^48–50^ The release of each water molecule from the octahedral (*O*_h_) coordination sphere caused the 1D chain structure to deform around the Zn^2+^ ion. Under ambient air, **1** converted to **1a** by capturing atmospheric moisture.^38^

### Crystal melting and glass formation

**Fig. 2:**
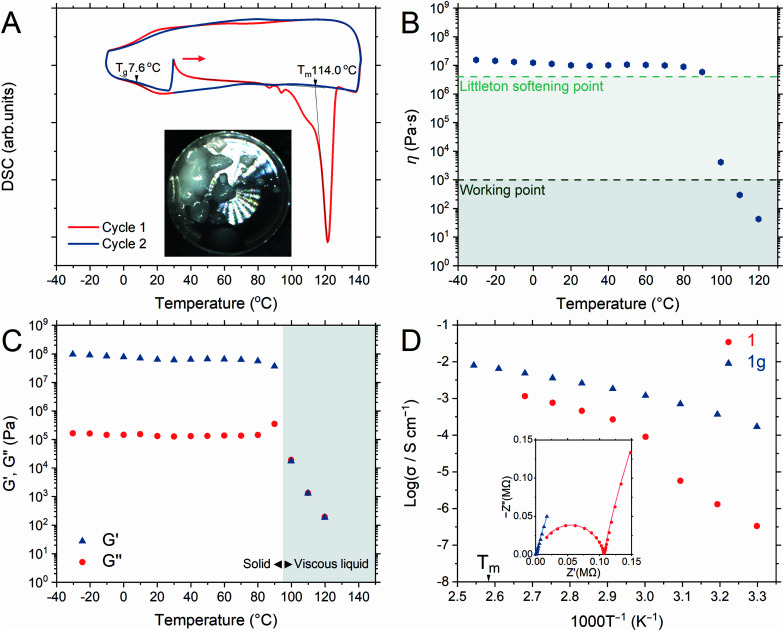
(A) First- (blue) and second-cycle (red) DSC profiles of 1 from −10 to 140 °C (begin with a heating step from 30 °C). The inset shows a photo of **1m** at 140 °C. (B) Temperature-dependent viscosity of **1g**. (C) DMA of **1g** from −30 to 120 °C (heating rate of 2 °C min^−1^). The storage (G′) and loss (G′′) moduli were marked as (▲) and (●), respectively. (D) Arrhenius plots of the anhydrous conductivity of **1** (●) and **1g** (▲) under an Ar atmosphere. The inset shows the Nyquist plot of **1** (●) and **1g** (▲) at 50 °C.

Differential thermal analysis of **1a** by TGA (Fig. S4†) showed two endothermic peaks due to the release of coordinated water and crystal-to-liquid transformation and only the latter peak was observed in **1**. DSC of **1** (Fig. 2A) showed an endothermic peak with an onset melting point (*T*_m_) of 114 °C. Two minor endothermic peaks before that of the *T*_m_ were assigned to the dehydration of adsorbed water during the measurement setup.^33^ The *T*_m_ of **1** was 50 °C lower than that of the isostructure, [Zn_3_(H_2_PO_4_)_6_(H_2_O)_3_](benzimidazole), as BTA exhibits lower *T*_m_ and p*K*_a_ values than those of benzimidazole.^31,33^ Additionally, no significant weight loss was seen at 120 °C after 12 h, confirming a stable liquid state (Fig. S6†). The liquid/molten state of **1** is henceforth referred to as **1m**. The first cooling process in DSC confirmed the vitrification of **1m** to a glassy state of **1** (denoted as **1g**) that demonstrated a glass transition temperature (*T*_g_) of 7.6 °C, exhibiting no Bragg diffraction, and was categorized as melt-quenched glass (MGQ) (Fig. S7†).^34^

DMA and viscosity evaluation of **1g** further determined its processing ability, where its viscosity (Fig. 2B) was observed above the Littleton softening point (10^6.6^ Pa s) from −30 to 90 °C until it sharply decreased below the working point regime (10^3^ Pa s) above 100 °C. The working point defines the viscosity regime in which the viscosity of a substance is equivalent to that of soda–lime–silica glass above 1100 °C (suitable for industrial forming processes).^51^ The storage modulus (*G*′) dominated the loss modulus (*G*′′) from −30 to 90 °C, verifying the solid character of **1g** (Fig. 2C). Immediate reduction of *G*′ at 100 °C represents the softening of **1g**, and the *G*′/*G*′′ crossover indicates the range in which **1g** starts to behave like a viscous liquid.^31,52^

### Anhydrous H^+^ conductivity

We measured the H^+^ conductivity of **1** and **1g***via* variable-temperature alternating current (AC) impedance under an Ar atmosphere to exclude the influence of water molecules (Fig. 2D and S9†). The Nyquist plots were fitted with a single impedance response corresponding to the bulk resistance without the grain-boundary region.^52,53^ The conductivity of **1** was measured from 30 to 100 °C, where the crystalline phase of **1** was preserved. We observed conductivity values of 3.3 × 10^−7^ S cm^−1^ and 9.0 × 10^−5^ S cm^−1^ at 30 and 60 °C, respectively. The conductivity value increased rapidly upon heating, reaching 1.2 × 10^−3^ S cm^−1^ at 100 °C. The activation energy of **1** from 30 to 60 °C was 1.22 eV. Above 60 °C, the Arrhenius plot flattened and the activation energy reduced to 0.57 eV. Utilizing BTA with its low p*K*_a_ in **1** provided higher conductivity values than those of the isostructure [Zn_3_(H_2_PO_4_)_6_](HBim) at 30 °C (1.2 × 10^−7^ S cm^−1^) and 60 °C (1.5 × 10^−5^ S cm^−1^).^38^

To highlight the advantage of glass transformation on ionic conductivity, we prepared a monolith **(1g**) *via* melt-quenching directly into the electrochemical cell for impedance analysis. Upon the crystalline-to-glassy state transformation, only the bulk impedance response pattern was obtained (Fig. S9†) and it was identical to that of **1a** in the higher temperature range. The Arrhenius plot (Fig. 2D) shows two different activation energy regimes: 0.59 eV between 30 and 60 °C and 0.39 eV from 60 to 120 °C. At 30 °C, **1g** exhibited a conductivity value of 3.3 × 10^−4^ S cm^−1^, which increased to 4.9 × 10^−3^ S cm^−1^ and 6.5 × 10^−3^ S cm^−1^ at 100 and 110 °C, respectively. A conductivity value of 8.0 × 10^−3^ was achieved at 120 °C (molten state, **1m**). Long-term conductivity retention was also evaluated. After 12 h, less than 4% and 10% loss in conductivity was observed at 100 and 120 °C, respectively (Fig. S10†). The contribution of the ions of interest to the total current can be distinguished *via* the H^+^ transport number (transference number) measurements.^52,54^ The transport numbers of most aqueous and ionic liquid electrolytes are lower than 0.6.^54–56^ The transport number of **1m** was elucidated *via* electromotive force (EMF) measurements, which were conducted for different hydrogen partial pressure (−ln(*P*_1_/*P*_2_) values of 0.22, 0.51, 0.69, 0.92, and 1.61) at 120 °C (Fig. S11†).^57^ According to eqn S1 (ESI),† the H^+^ transference number of **1g** is 1.0, indicating an ideal single-ion H^+^ conductivity. The absence of anion mobility suggests that the coordination networks are retained even in the molten state.^31,58^

### Proton dynamics in **1** and **1g**

**Fig. 3:**
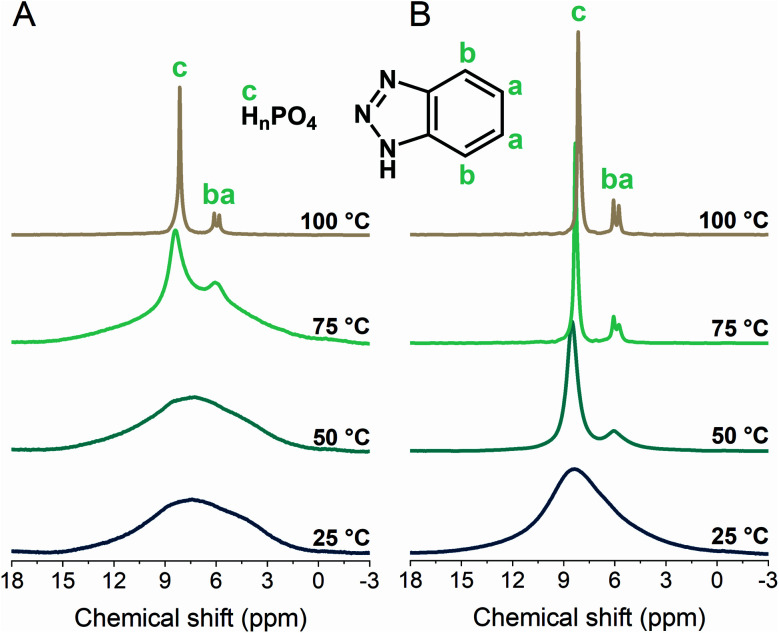
Variable-temperature ^1^H magic-angle spinning (MAS) solid-state NMR spectra (MAS 8 kHz) of (A) **1** and (B) **1g** from 25 to 100 °C.

The H^+^ conductivity would be dominated by either the phosphate or BTA dynamics; therefore, we utilized variable-temperature ^1^H MAS solid-state NMR to study their mobilities (Fig. 3). The peaks from 8.1–8.5 and 5.8–6.1 ppm were assigned to the phosphate and BTA H^+^, respectively.^44,52^ The substantial narrowing and intensifying of the peaks between 50 and 75 °C suggested a significant increase in both the phosphate and BTA dynamics. The molecular motion of BTA initiates at the temperature above 50 °C as the BTA peaks are barely distinguishable at 25 and 50 °C (Fig. 3A).^38^ The H^+^ mobilities of **1g** and **1** were compared at 25 and 50 °C as well as at 50 and 75 °C, where the narrower and more intense peaks of **1g** demonstrated its higher H^+^ mobility than that of **1** (Fig. 3B). This higher degree of H^+^ mobility was promoted by a disordered structure formed in **1g**. Furthermore, the BTA dynamics were observable in **1g** even at temperatures lower than 60 °C, which agrees with the impedance response and lower activation energy of **1g**. Additionally, hydrogen-bond formations are indicated by downfield shifts.^59^

### Electrode–electrolyte interface

**Fig. 4:**
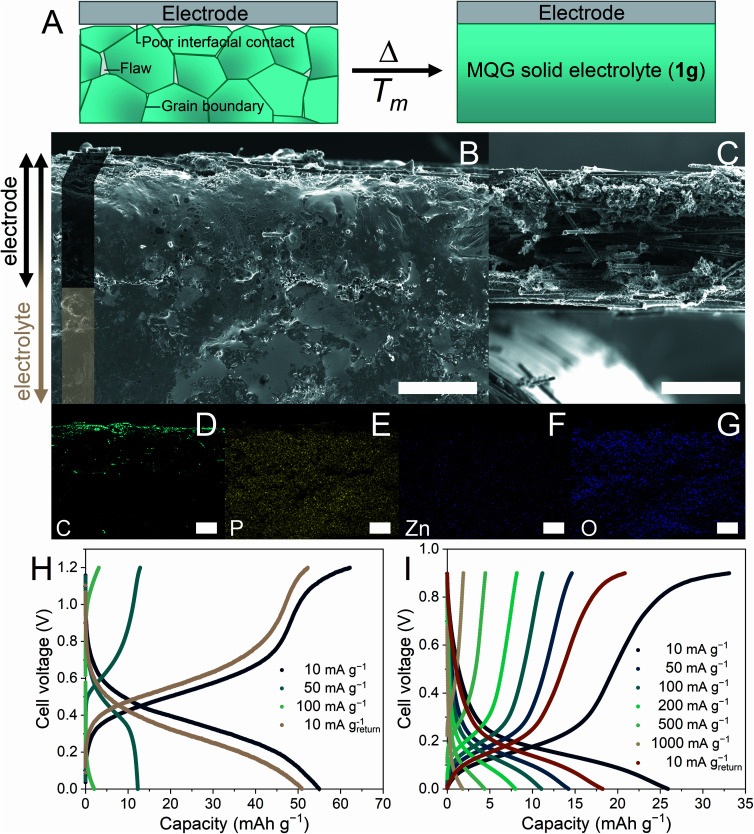
(A) Schematic representation of the interfaces/flaws within the polycrystalline solid electrolyte (left) and MQG solid electrolyte proposed in this work (**1g**). Cross-sectional scanning electron microscopy (SEM) images (×150 magnification) of (B) the electrode–solid-state electrolyte interface (**CF–1g**) and (C) **CF** electrode. Scale bar = 150 μm. Energy-dispersive X-ray (EDX) mapping for (D) C, (E) P, (F) Zn, and (G) O. Scale bar = 100 μm. Full-cell charge–discharge profiles utilizing **1g** as a solid-state electrolyte at (H) 25 °C and (I) 100 °C.

Discontinuities along the heterogeneous interface inhibit practical applications of solid-state electrolytes.^17^ Therefore, we are interested in the H^+^-conductivity integration and moldability of **1g** as a grain boundary-free immersive solid electrolyte (Fig. 4A). Additionally, the lower *T*_m_ of **1** would prevent the anode/cathode materials from degrading during the fabrication process.^8,9,12,13^ A carbon fiber (CF) electrode was pressed to **1m** at 120 °C and quenched to room temperature to provide the electrode–electrolyte interface (**CF–1g**). Cross-sectional scanning electron microscopy (SEM) images of **CF–1g** were collected (Fig. 4B, S12A, and B†), where neither a distinguishable solid–electrolyte interface nor grain boundaries were observed.^60^ Optimum contact between the CF layer and **1g** domain was achieved as **1m** can penetrate the CF, generating a fully immersed environment. Fig. 4C shows a cross-sectional SEM reference image of the pristine **CF**. Energy-dispersive X-ray (EDX) mapping (Fig. 4D–G) further elucidated the position of the **CF** electrode (intense C) with homogeneously distributed Zn, P, and O signals. To amplify the benefits of melt-quenching glass, we re-examined the morphological alteration of **CF–1g** after recrystallization. **1g** undergoes the recrystallization process upon humidity exposure and transforms back to **1a**. As confirmed by PXRD (Fig. S13†), atmospheric humidity (65% relative humidity) at room temperature (25 °C) is sufficient for the recrystallization to occur within 4 h. Fig. S12C and D† revealed grain boundaries and fractures formed throughout the recrystallized **1g** matrix, especially in the region where the CF and **1g** co-exist.

### Solid-state H^+^ battery under anhydrous conditions

Adequately high H^+^ conductivity, single-ion conductivity, low processing temperatures, and thermal/electrochemical stabilities motivated us to apply **1g** as a solid electrolyte for H^+^ batteries. MoO_3_ and Cu^II^[Fe^III^(CN)_6_]_2/3_·4H_2_O (CuFe-TBA) were selected as a model cathode and anode, respectively.^10,12^ As a reference, we also evaluated a full-cell configuration in 2 M H_2_SO_4_ solution at 25 °C. It exhibited a discharge capacity of 35.8 mA h g^−1^ at 100 mA g^−1^ (Fig. S14†). The specific capacity was calculated based on the cathode mass. The distance between the electrodes was *ca.* 1 cm.^12^ The solid-state H^+^ battery was prepared by immersing both electrodes (1 cm separations) in **1m** at 120 °C under an Ar atmosphere, where subsequent quenching to room temperature gave the **1g** electrolyte. Fig. 4H and S15A† show the charge–discharge profiles (from 0 to 1.2 V) and rate performance evaluation of solid-state H^+^ batteries under an Ar atmosphere utilizing the **1g** electrolyte at 25 °C. The highest discharge capacity was 55.4 mA h g^−1^ at 10 mA g^−1^. Another advantage the **1g**-electrolyte system has over the aqueous system is its large operating-temperature range. The elevated-temperature H^+^ battery was evaluated at 100 and 110 °C under an Ar atmosphere (Fig. 4I and S15C†) and the redox potentials of both electrodes reduced, corresponding to the change in free energy.^61,62^ As shown in Fig. S15B and D,† rate performances improved significantly as the ionic conductivity of **1g** was enhanced.^2^ In a high-temperature regime, electrodes would show an excessive self-discharge as well as a thermal structural distortion limiting the protonation/deprotonation processes, causing a net loss of capacity. For instance, in a Li-ion battery, capacity fading was observed in Li_3_V_2_(PO_4_)_3_ as elevated temperature promotes a larger structural distortion between Li_3_V_2_(PO_4_)_3_ and V_2_(PO_4_)_3_ limiting the re-insertion of Li^+^.^63^ Additionally, 76% of the original capacity was retained after 1000 cycles of the charge–discharge process at 110 °C (Fig. S16†). We also attempted to demonstrate a solid-state H^+^ battery using crystalline **1** with a similar configuration and an identical anode and cathode. However, charging and discharging processes were not possible at 25 °C nor under low-current (10 mA g^−1^) conditions, even though the thickness of this electrolyte was ten times smaller than that of the **1g** electrolyte. This emphasizes the importance of interface engineering that endows soft glass materials with high H^+^ conductivity and moldability.^17,20,21^

## Conclusions

We synthesized a new H^+^ conductive CP, [Zn_3_(H_2_PO_4_)_6_(H_2_O)_3_](BTA), where the dehydrated state (**1**) integrated promising anhydrous H^+^ conductivity (1.2 × 10^−3^ S cm^−1^ at 100 °C) and relatively low melting point (114 °C). The melt-quenched glass of **1** (**1g**) enhanced the H^+^ dynamics of both phosphate and BTA, resulting in a H^+^ conductivity value of 8.0 × 10^−3^ S cm^−1^, a H^+^ transference number of 1.0, and a viscosity of 42.8 Pa s at 120 °C. The coexistence of high conductivity, transport number, and moldability of **1g**, as well as its flawless interface, encouraged us to implement it in solid-state H^+^ battery applications. A solid-state H^+^ battery with an operating temperature range above room temperature (25–110 °C) was demonstrated for the first time. The tuning capability of the CP glass H^+^ conductivity, working temperature, and softness could provide H^+^ batteries with wider applications.

## Author contributions

S. H. designed the project, and N. M. and A. Y. synthesized the compounds. S. K. collected and analyzed solid-state NMR measurements. N. M. collected and analyzed SC-XRD, PXRD, TGA, SEM, DSC, ICP-ES, DMA, FTIR, conductivity and transport number measurements and battery evaluation. S. H. and N. M. wrote the paper.

## Conflicts of interest

There are no conflicts to declare.

## Supplementary Material

SC-012-D1SC00392E-s001

SC-012-D1SC00392E-s002
