# Supplementary material for: Proton-conductive coordination polymer glass for solid-state anhydrous proton batteries
Source: Chem Sci. 2021 Mar 12;12(16):5818–24. doi: 10.1039/d1sc00392e (PMC8179665; doi:10.1039/d1sc00392e)
Supplement: SC-012-D1SC00392E-s001 [file SC-012-D1SC00392E-s001.pdf]

Electronic Supplementary Material (ESI) for Chemical Science.
This journal is © The Royal Society of Chemistry 2021

Supplementary Information for

Proton-Conductive Coordination Polymer Glass for Solid-State Anhydrous Proton Batteries

Nattapol Ma,[a] Soracha Kosasang,[b] Atsushi Yoshida [a] and Satoshi Horike *[acde]

[a] Department of Synthetic Chemistry and Biological Chemistry, Graduate School of Engineering, Kyoto University, Katsura, Nishikyo-ku, Kyoto 615-8510, Japan
[b] Department of Chemical and Biomolecular Engineering, School of Energy Science and Engineering, Vidyasirimedhi Institute of Science and Technology, Rayong, 21210, Thailand
[c] AIST-Kyoto University Chemical Energy Materials Open Innovation Laboratory (ChEM-OIL), National Institute of Advanced Industrial Science and Technology (AIST), Yoshida-Honmachi, Sakyo-ku, Kyoto 606-8501, Japan
[d] Institute for Integrated Cell-Material Sciences, Institute for Advanced Study, Kyoto University, Yoshida-Honmachi, Sakyo-ku, Kyoto 606-8501, Japan
[e] Department of Materials Science and Engineering, School of Molecular Science and Engineering, Vidyasirimedhi Institute of Science and Technology, Rayong, 21210, Thailand

*Email: horike@icems.kyoto-u.ac.jp (S.H)

Table of Contents

1. Structural determination of **1a** S2
2. Experimental Section S2 – S4
3. Supplementary figures and table S5 – S13
4. References S14

1. Structure Determination of 1a

Formula	$[Zn_3(H_2PO_4)_6(H_2O)_3]\cdot(C_6H_5N_3)$
MW / g mol^{-1}	880.02
Crystal System	Triclinic
Space Group	*P*-1
Z	2
a / Å	7.47650(10)
b / Å	14.1513(3)
c / Å	15.0795(4)
α / °	115.507(3)
β / °	99.363(2)
γ / °	99.595(2)
V / Å^3	1369.63 Å^3
T / K	100
R-factor / %	3.11
w*R* / %	8.51
GOF	1.135

2. Experimental section

2.1 Synthesis of $Zn_3(H_2PO_4)_6(H_2O)_3$](1,2,3-benzotriazole) (1a). The ZnO (99.99% trace metals basis), phosphoric acid (85% in H_2O), and 1,2,3-Benzotriazole (>98.0%, HPLC) were purchased from Sigma Aldrich, Wako pure chemical, and Tokyo Chemical Industry, respectively. All chemicals were used without further purification. Zinc oxide (1281.7 mg, 15.75 mmol), 1,2,3-Benzotriazole (625.4 mg, 5.25 mmol), phosphoric acid (31.5 mmol, 2163 µL) were added to a 25 mL Teflon jar with three steel-cored 10 mm Teflon balls. The mixer was milled for 60 min at 25 Hz, using Retsch MM 300 mixer mill. The product was evacuated at room temperature for 3.5 h to obtained **1a** in pure phase and kept in an Ar-filled glovebox to prevent adsorption of excess moisture. A single crystal of **1a** was collected by evaporating an identical mixture with excess phosphoric acid in ethanol solvent at 60 °C. **1** is obtained by evacuating **1a** at 80 °C for 12 h. Crystallographic data for the **1a** has been deposited with the Cambridge Crystallographic Data Centre, CCDC, depository number 2044808.

2.2 Material characterizations. The single-crystal X-ray diffraction SC-XRD measurement was collected with a Rigaku XtaLab P200 diffractometer and a Dectris Pilatus 200K system at 302 K. The system is equipped with A MicroMax007 HF/VariMax rotating-anode X-ray generator with confocal monochromatized MoKα radiation. The collected data was solved and refined by full-matrix least-squares refinement using the SHELXL 2016/4. Powder X-ray diffraction (PXRD) patterns were collected using a Rigaku MiniFlex with CuKα anode.

Thermalgravimetric analysis (TGA) results were obtained using a Rigaku Thermo plus TG 8121 with a heating rate of 10 °C min^{-1} under flowing Ar. Scanning electron microscope (SEM) images were taken using a Hitachi SU5000 instrument after Osmium plasma-chemical vapor deposition for 5 s. CF sheet was hand-pressed to **1m**, followed by quenching at room temperature. Cross-section samples were cut at 77 K. Differential Scanning Calorimetry (DSC) was collected using Hitachi DSC 7020 in an N_2 atmosphere (Pt crucible). Inductively coupled plasma emission spectroscopy (ICP-ES) were measured using Shimadzu ICPE-9000. The sample was digested in nitric acid with yttrium internal standard. ^{31}P MAS Solid-state NMR (600 MHz) was performed on a JNM-ECZ600R (JEOL RESONANCE Inc.) solid-state NMR spectrometer at 14.1 T at room temperature at 20 kHz (3.2 mm rotor). Variable temperature ^{1}H MAS Solid-state NMR was measured by the single pulse with a relaxation time of 300 s at an 8 kHz spinning rate from 25 to 100 °C (^{1}H Resonance frequency of 600.2 MHz). The spectra were processed with Delta software (JEOL RESONANCE Inc). Dynamic mechanical analysis and viscosity measurement were evaluated via a rotational parallel-plate rheometer (Rheosol-G5000, UBM Co., Ltd.) under N_2 flow, applying oscillatory strain of 1 Hz. IR spectra were obtained with a Thermo Scientific Nicolet Summit FT-IR equipped with a diamond ATR accessory. Note, measurements were performed under ambient atmosphere. Conductivity measurements were performed using impedance spectroscopy technique. Crystalline sample (ca. 50 mg) was pressed at 500 kgf for 2 min using 5 mm die. The pellet was sandwiched between two gold electrodes. The impedance measurements were performed in Ar-filled glovebox using impedance and gain-phase analyzer (Solartron SI 1260 Impedance/Gain-Phase analyzer) over the frequency range 1 Hz - 1 MHz with an input voltage amplitude of 30 mV. Impedance data of glassy state were collected using a BioLogic VSP-300 with a modified stainless coin cell setup and Pt electrode EC Frontier. Sample was filled in liquid state and cool down to room temperature (melt-quenching). Collected data were analyzed using ZView software via equivalent circuit fitting.

2.3 Proton transport number. Proton transport number or transference number (t_{H^+}) was evaluated via the electromotive force (EMF) measurements to distinguish the contribution of H^+ on overall conductivity. A 2 × 2 cm membrane sheet (Omnipore™ Merck, 10.0 μM) was impregnated with the **1m** at 120 °C then quenching to room temperature. The membrane was then sandwiched between two platinum-coated carbon electrodes (1.5 mg cm^{-2}, Ø = 7 mm, Chemix Co. Ltd.), then inserted into a single cell with straight gas flow channels. On one side H_2/Ar gas (3.99 vol%) was fed uniformly at 100 SCCM. On the other side, variable partial pressures were achieved by mixing H_2/Ar gas (3.99 vol%) and N_2 (99.99995 vol%). Note, the gas flow was precisely controlled by mass flow controllers (SEC-E40, Horiba, Ltd). The total mass flow of 100 SCCM was maintaining for both sides. The cell was then heated to the target temperature and stabilized for 1 h before evaluation. The EMF between 2 sides was measured under various H_2 partial pressure (P_2) on equilibrium for 5 min ($\ln(P_1/P_2)$ = 0.22, 0.51, 0.69, 0.92, and 1.61). The t_{H^+} was calculated via the following equation:

$$E = t_{H^+}\frac{RT}{2F}\ln\left(\frac{P_1}{P_2}\right) \qquad (1)$$

where, E, T, R, F, P_1, and P_2 represent EMF, temperature, the gas constant, Faraday constant, partial pressure of H_2 at constant, and variable side, respectively.

2.4 Proton batteries evaluation. CuFe-TBA ($Cu^{II}[Fe^{III}(CN)_6]_{2/3}\cdot 4H_2O$) and MoO_3 (99.5%, Nacalai) were selected as cathode and anode, respectively.[1-4] CuFe-TBA was prepared by an established method[2, 5]. To fabricate the electrode (both anode and cathode), 70 wt% active material, 20 wt% conductive Ketjen black, and 10 wt% polyvinylidene fluoride (PVDF) binder were ground in a mortar with a small amount of N-Methyl-2-pyrrolidone.

The homogeneous slurry was then cast on carbon fiber paper (SGL Carbon) and dried in an oven at 60 °C.[2, 4, 6] The active mass loading for CuFe-TBA is ca. 1.5 mg cm^{-1}. An excess amount of MoO_3 was utilized and specific capacity was calculated based on cathode mass. We first confirm the electrochemical performance of CuFe-TBA and MoO_3 in the aqueous system (2 M H_2SO_4) via a three-electrode configuration (Fig. S13 A and B). As-prepared CuFe-TBA or MoO_3, Pt wire, and Ag/AgCl (3 M NaCl) were utilized as a working electrode, a counter electrode, and a reference electrode, respectively. After the half-cell redox reaction was confirmed, a full-cell evaluation (two-electrode) of CuFe-TBA cathode and MoO_3 anode was then conducted in 2 M H_2SO_4. Anode and cathode were placed 1 cm apart without using any separator. No distinct performance decay due to the dissolution of the PVDF binder was observed. A full-cell was charged to 1.2 V and discharge to 0 V with at 100 mA g^{-1} to 2000 mA g^{-1} (Fig. S13 C and D). Note, CuFe-TBA undergoes a charging process before full cell fabrication. The distance between two electrodes was kept ca. 1 cm. The solid-state proton battery was prepared by filling 5 ml cell with **1** (powder). Both electrodes were placed with the 1 cm distance between electrodes. The cell was then heated to the melting point of the 1. The temperature was held for 30 min to ensure a homogeneous liquid state. The cell was then cool to room temperature to obtain a solid-state proton battery. Both two-electrode and three-electrode evaluations were performed using BioLogic VSP-300 with galvanostatic charge-discharge (GCD chronopotentiometry). Measurement temperature of the solid-state battery was controlled using a Sibata DBH-1000 heater. Before each measurement, the target temperature was held constate for at least 1 h. No electrolyte decomposition was found within the operating range of full-cell proton batteries. As a reference, pelletized **1** with a thickness of 1 mm (17 mm diameter) was sandwiched between similar cathode and anode. A CR2032 coin cell was fabricated and tested at 1 mA g^{-1}.

3. Supplementary Figures

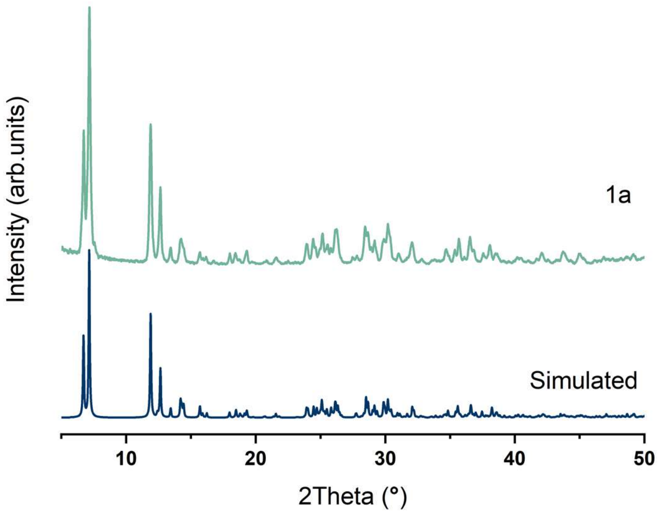

Fig. S1 PXRD patterns of **1a** (green) and simulated PXRD patterns (blue).

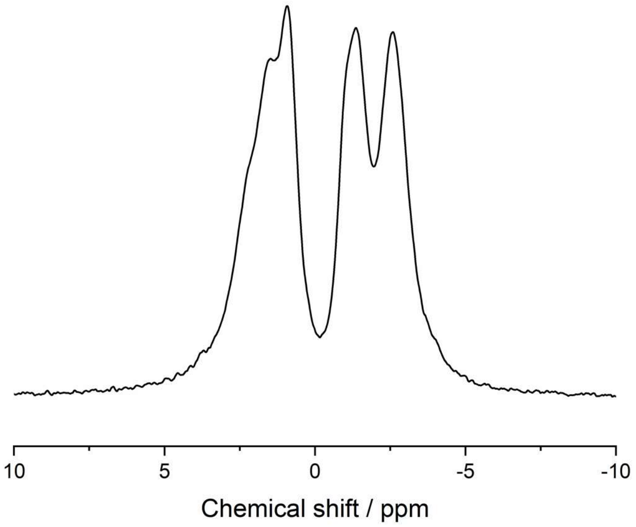

Fig. S2 ^{31}P Magic-angle spinning (MAS) NMR spectra of **1a** with a rotation rate of 20 kHz.

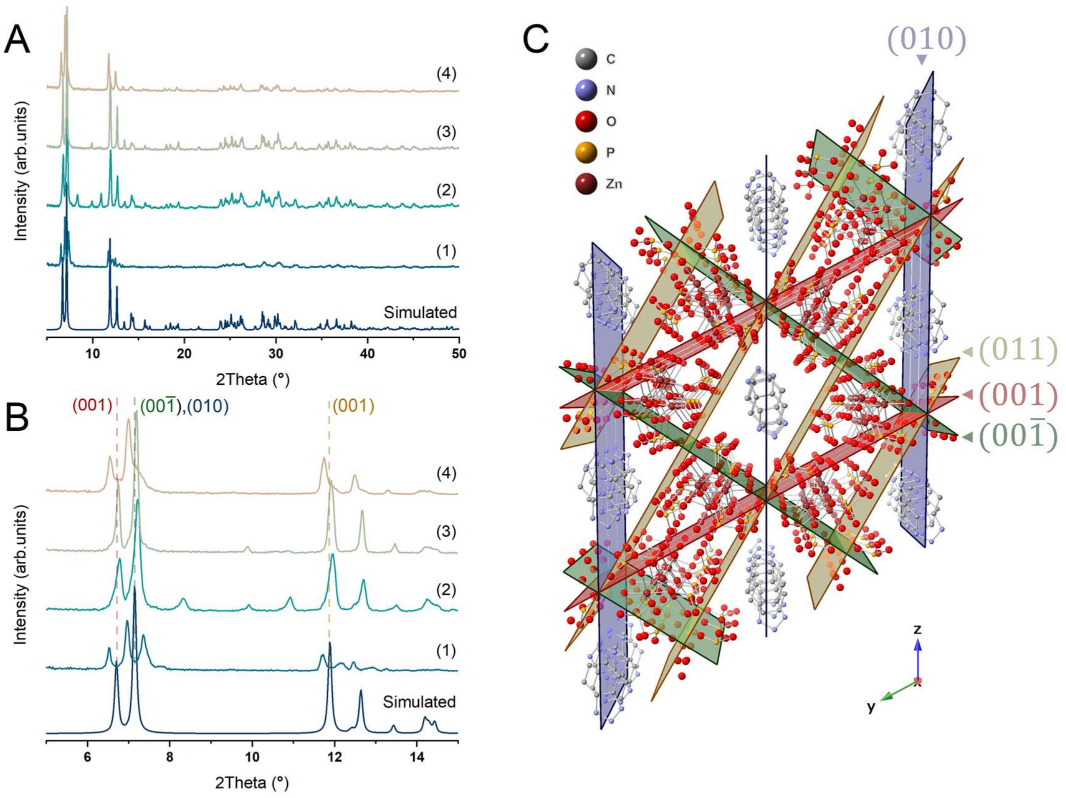

Fig. S3 PXRD pattern (A) between 5 ° to 50 ° and (B) between 5 ° to 15 °, represent following condition: Milling product from the stoichiometric ratio of Zinc oxide, 1,2,3-Benzotriazole, and phosphoric acid before vacuum drying (1). After 12 h of vacuum drying at room temperature (2), then left in humid air for 3h (3) and 12 h (4).

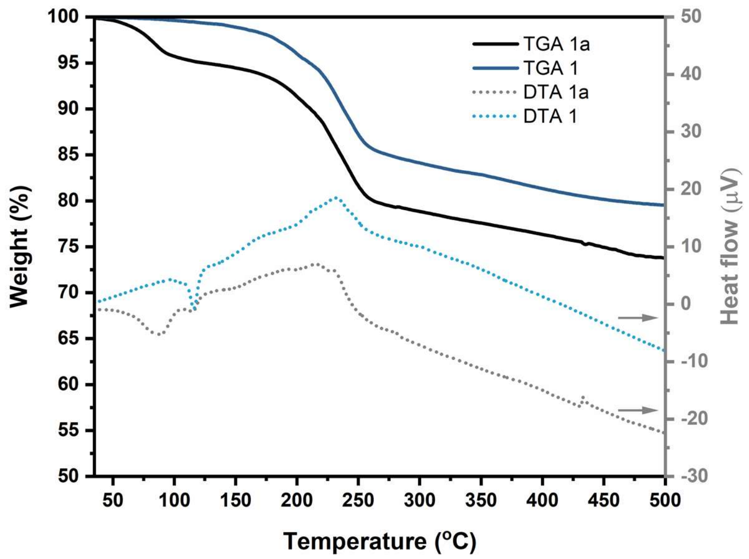

Fig. S4 TGA and DTA profile of **1a** and **1** from 40 to 500 °C (10 °C min^{-1}).

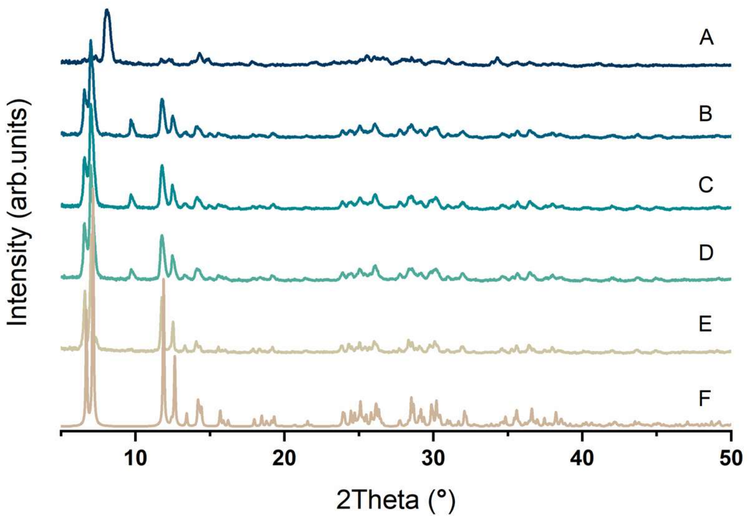

Fig. S5 PXRD of **1** after humid air exposure for (A) 0 min, (B) 15min, (C) 30 min, (D) 90 min, (E) 4 h, as well as (F) the simulated pattern of **1a**.

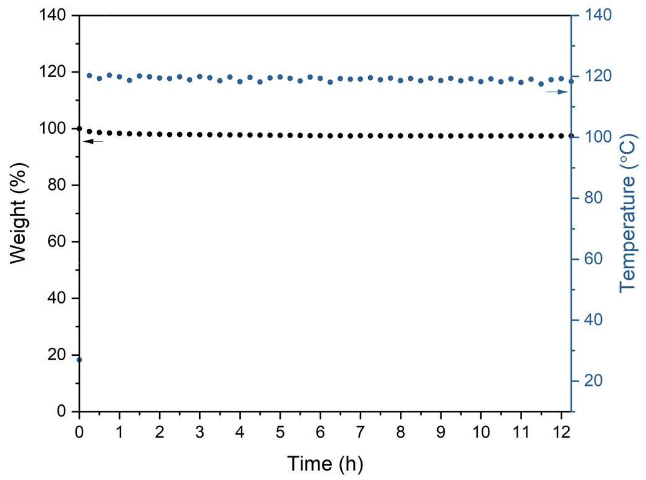

Fig. S6 Constant temperature TGA of **1m** at 120 °C.

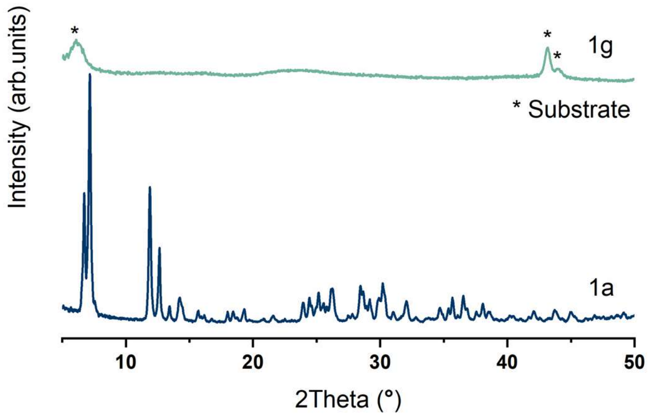

Fig. S7 PXRD patterns of **1g** (green) and **1a** (blue). Ar cell background peaks represent as (*).

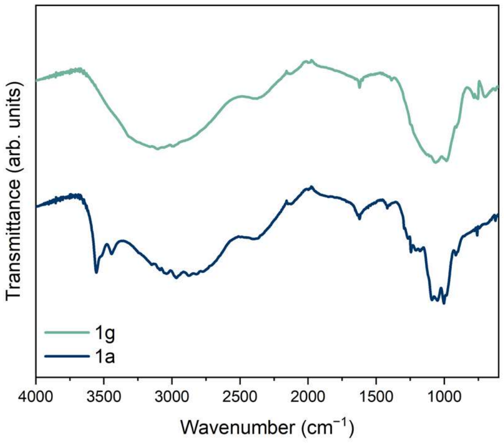

Fig. S8 IR spectra of **1g** (green) and **1** (blue).

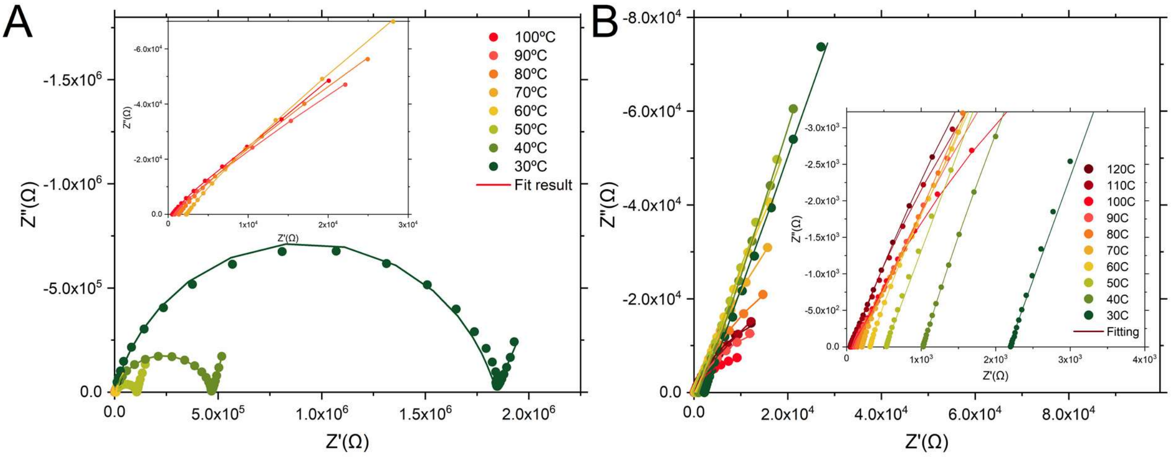

Fig. S9 Nyquist plots of (A) **1** from 30 to 100 °C and (B) **1g** from 30 to 120 °C. Two types of equivalent circuit models were applied to fit the impedance in 2 different temperature ranges. Between 30 to 50 °C, **1** exhibits two series (RQ) impedance response corresponding to bulk resistance and electrode-electrolyte interface, respectively. Above 50 °C, R(RQ) impedance model was utilized. Note, R and Q represent resistor and constant phase element.[7, 8]

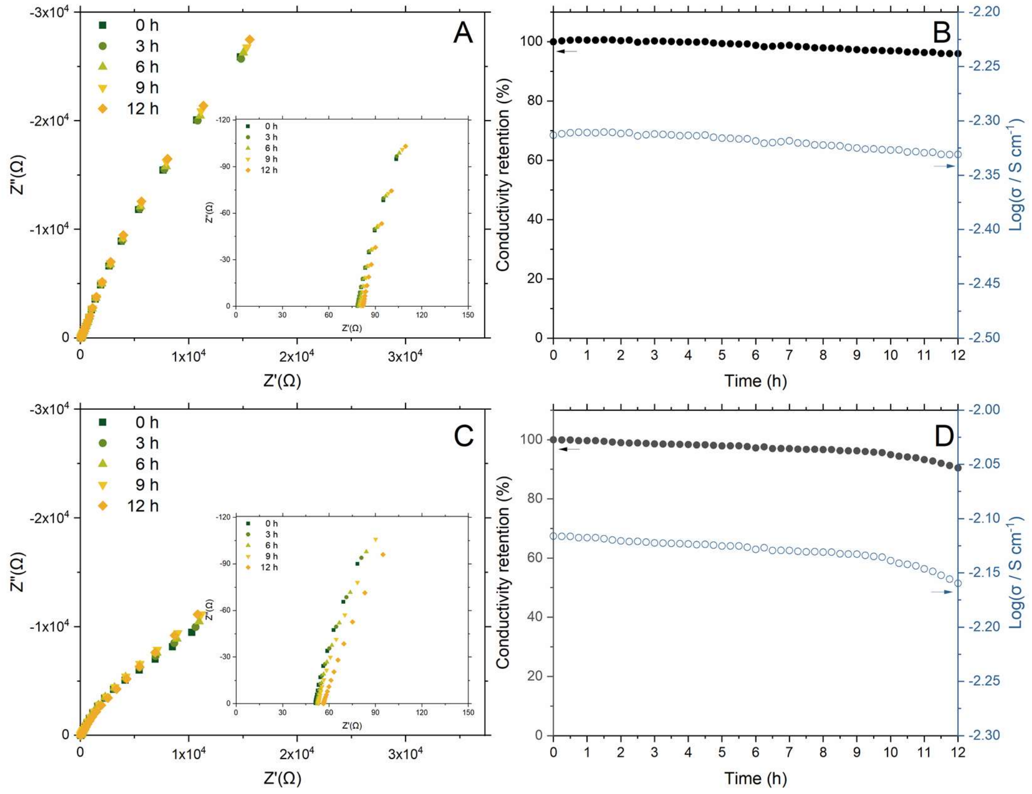

Fig. S10 Nyquist plots and Long-term proton conductivity retention of **1g** for 12 h at 100 °C (A) and (B) as well as 120 °C (C) and (D).

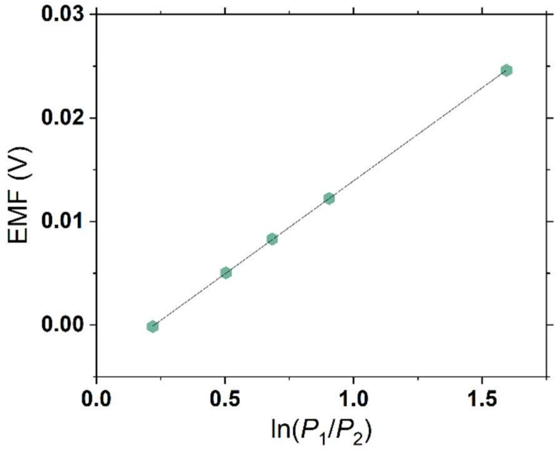

Fig. S11 Electromotive force (EMF) measurement of **1g** at 120 °C with experimental data fitting line.

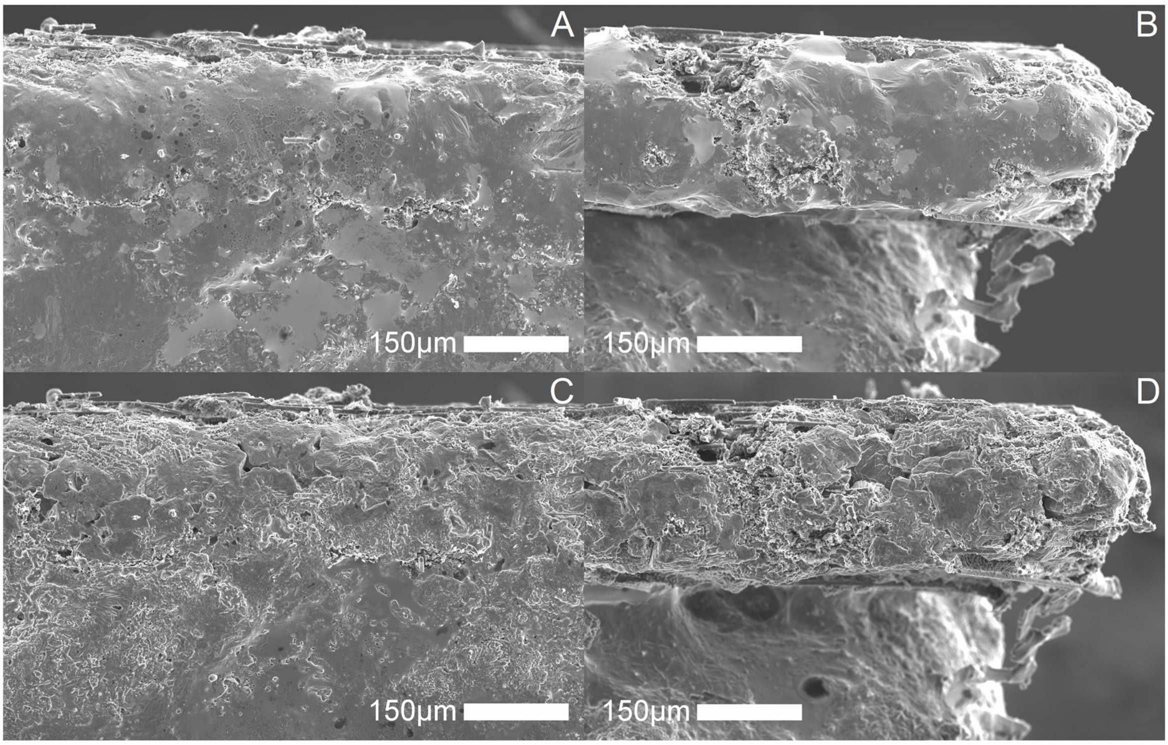

Fig. S12 Cross-section SEM images (x150 magnification) of the electrode-electrolyte interface of the as-prepared sample at (A) mid-section and (B) edge. The as-prepared sample was then left in the humid air for 4 h and keep in the Ar-filled glove box for 20 h to demonstrate the recrystallization of **1g**. SEM images recrystallized sample at (C) mid-section and (D) edge.

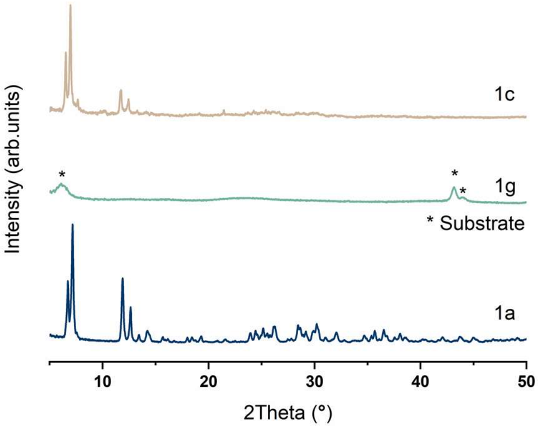

Fig. S13 PXRD patterns of the recrystallized sample (beige), **1g** (green), and **1a** (blue). Ar cell background peaks represent as (*).

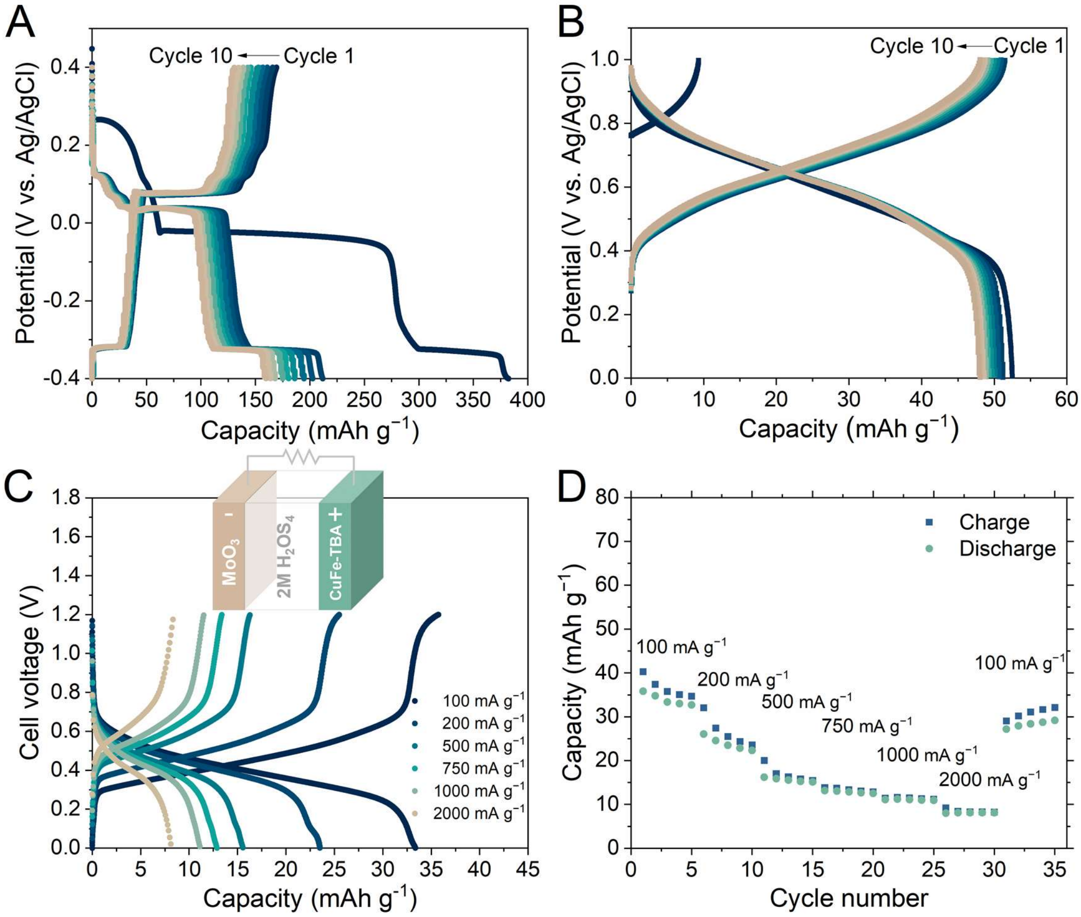

Fig. S14 GCD profiles at various specific current in 2 M H_2SO_4 of (A) MoO_3, (B) CuFe-TBA, and (C) Full cell batteries (inset demonstrates cell configuration). (D) Full cell capacity at various applied current.

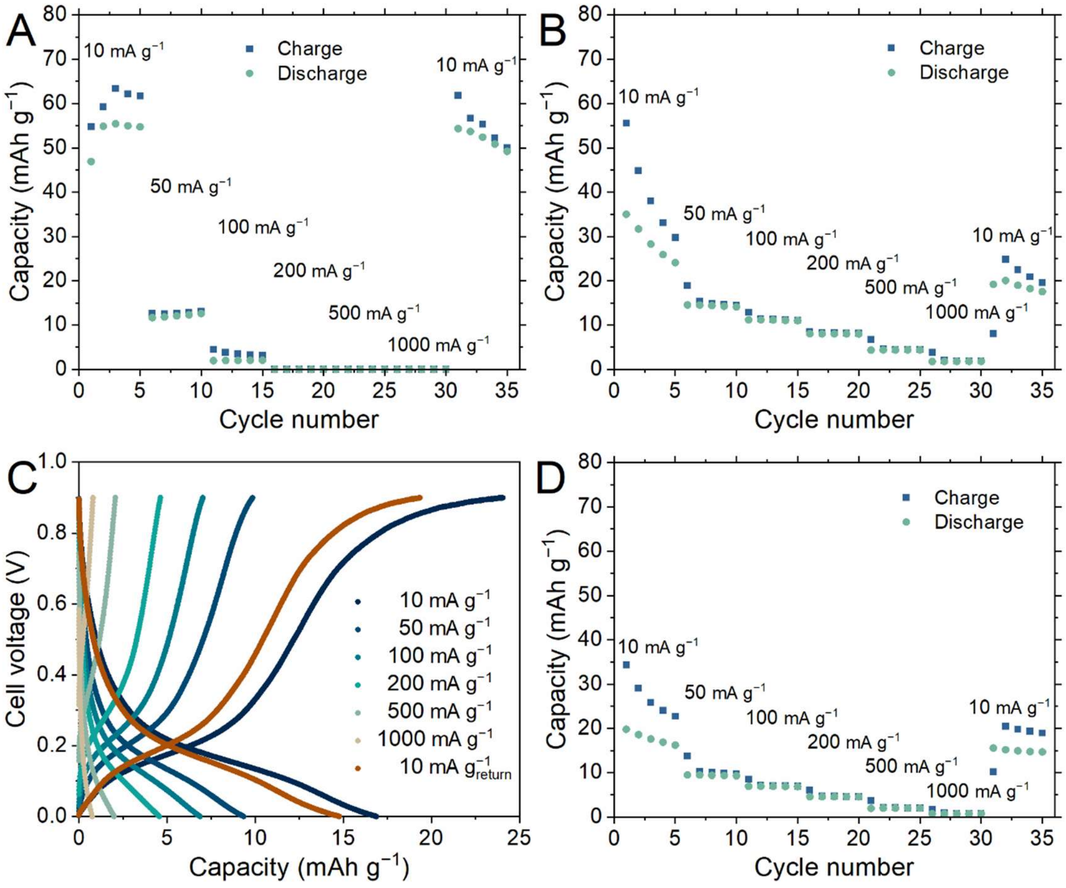

Fig. S15 Rate capacity utilizing **1g** as a solid-state electrolyte at (A) 25 °C and (B) 100 °C. Full cell charge-discharge profiles (C) and rate capacity (D) at 110 °C.

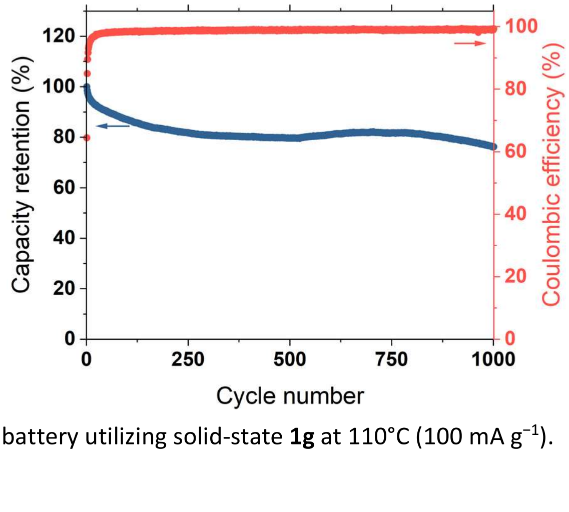

Fig. S16 Stability of full cell battery utilizing solid-state **1g** at 110°C (100 mA g^{-1}).

Table 1. Full-cell proton battery of representative systems.

Anode<br>Cathode	Electrolyte<br>(state)	Temperature range<br>(Ionic conductivity)	Rate performance*	Estimated Discharge Voltage	Cycling stability	Ref.
PEDOT-AQ<br>PEDOT-BQ	2-fluoropyridinium triflate-2-fluoropyridine (ionic liquid)	22 ˚C (n/a)	103 and 25 mAh g^{-1}<br>(70 mA g^{-1} and 120 A g^{-1})	0.55 V	44.4% after 125 cycles	9
WO_3<br>CuFe-TBA	Aqueous 2.0 M H2SO4 (liquid)	25 ˚C (n/a)	50 mAh g^{-1}<br>(0.5 A g^{-1}, −70 ˚C)	1.0 V	74% after 1000 cycles	2
MoO_3<br>MnO2@GF	Aqueous 2 M H_2SO_4<br>+ 2 M $MnSO_4$<br>(solid below −40 ˚C)	−78 ˚C to 25 ˚C<br>(2.66 mS cm^{-1} at −70 ˚C<br>139.9 mS cm^{-1} at 25 ˚C)	ca. 119 mAh g^{-1}<br>(0.5 A g^{-1}, −70 ˚C)	1.5 and 1.14 V	100% after 100 cycles at −70 ˚C	10
pEP(NQ)E<br>pEP(QH_2)E	Aqueous 3.3 M (−24 ˚C) and 0.5 M (22 ˚C) H_2SO_4 (liquid)	−24 ˚C to 22 ˚C<br>(n/a)	60 and 40 mAh g^{-1}<br>(1.1 and 3 A g^{-1}, −24 ˚C)	0.3 V (−24 ˚C)<br>0.4 V (22 ˚C)	98% after 500 cycles at −24 ˚C	11
MoO_3<br>CuFe-TBA	Aqueous 9.5 M H_3PO_4 (liquid)	−88 ˚C to 25 ˚C<br>(0.11 S cm^{-1} at 25 ˚C)	28 and 49 mAh g^{-1}<br>(25 mA g^{-1}, −78 and 25˚C)	1.0 V	100% after 450 cycles at −78 ˚C	12
MoO_3<br>CuFe-TBA	1.0 M H_3PO_4 in MeCN (liquid)	25 ˚C<br>(0.50 mS cm^{-1})	48 mAh g^{-1}<br>(100 mA g^{-1})	0.6 V	ca. 48% after 100 cycles	13
MoO_3<br>ZnFe-TBA	1.0 M H_3PO_4 in MeCN (liquid)	25 ˚C<br>(0.50 mS cm^{-1})	48 mAh g^{-1}<br>(100 mA g^{-1})	0.7 V	ca. 79% after 100 cycles	13
MoO_3<br>CuFe-TBA<br>(This work)	$[Zn_3(H_2PO_4)_6(H_2O)_3]$(1,2,3-benzotriazole) glass (**1g**) (solid)	30 ˚C to 110 ˚C<br>(0.33 mS cm^{-1} at 30 ˚C<br>6.5 mS cm^{-1} at 110 ˚C)	55 mAh g^{-1}<br>(10 mA g^{-1}, 30 ˚C)<br>28 mAh g^{-1}<br>(10 mA g^{-1}, 100 ˚C)<br>18 mAh g^{-1}<br>(10 mA g^{-1}, 110 ˚C)	0.4 V (25 ˚C)<br>0.2 V (110 ˚C)	76% after 1000 cycles At 110 ˚C	This work

The abbreviations of PEDOT-AQ, PEDOT-BQ, WO_3, CuFe-TBA, MoO_3, MnO_2@GF, NiFe-TBA, ZnFe-TBA represent Poly(3,4-ethylenedioxythiophene) functionalized with anthraquinone, Poly(3,4-ethylenedioxy-thiophene) functionalized with benzonquinone, Tungsten trioxide, Copper hexacyanoferrate (Turnbull's blue analogs), Molybdenum trioxide, Manganese oxide on graphite felt, Nickel hexacyanoferrate (Turnbull's blue analogs), Zinc hexacyanoferrate (Turnbull's blue analogs), respectively.

*Rate performance was based on the mass of the limiting electrode.

References

1. X. Wang, Y. Xie, K. Tang, C. Wang and C. Yan, *Angew. Chem. Int. Ed.*, 2018, **57**, 11569-11573.
2. X. Wu, J. J. Hong, W. Shin, L. Ma, T. Liu, X. Bi, Y. Yuan, Y. Qi, T. W. Surta, W. Huang, J. Neuefeind, T. Wu, P. A. Greaney, J. Lu and X. Ji, *Nat. Energy*, 2019, **4**, 123-130.
3. X. Ji, *Energy Environ. Sci.*, 2019, **12**, 3203-3224.
4. X. Wu, S. Qiu, Y. Xu, L. Ma, X. Bi, Y. Yuan, T. Wu, R. Shahbazian-Yassar, J. Lu and X. Ji, *ACS Appl. Mater. Interfaces*, 2020, **12**, 9201-9208.
5. S. S. Kaye and J. R. Long, *J. Am. Chem. Soc.*, 2005, **127**, 6506-6507.
6. C. D. Wessells, R. A. Huggins and Y. Cui, *Nat. Commun.*, 2011, **2**, 550.
7. J. R. M. E. Barsoukov, *Impedance Spectroscopy Theory, Experiment, and Applications*, John Wiley & Sons, NJ, USA, 2nd edn., 2005.
8. T. Ogawa, K. Takahashi, S. S. Nagarkar, K. Ohara, Y.-l. Hong, Y. Nishiyama and S. Horike, *Chem. Sci.*, 2020, **11**, 5175-5181.
9. R. Emanuelsson, M. Sterby, M. Strømme and M. Sjödin, *J. Am. Chem. Soc.*, 2017, **139**, 4828-4834.
10. L. Yan, J. Huang, Z. Guo, X. Dong, Z. Wang and Y. Wang, *ACS Energy Lett.*, 2020, **5**, 685-691.
11. C. Strietzel, M. Sterby, H. Huang, M. Strømme, R. Emanuelsson and M. Sjödin, *Angew. Chem. Int. Ed.*, 2020, **59**, 9631-9638.
12. H. Jiang, W. Shin, L. Ma, J. J. Hong, Z. Wei, Y. Liu, S. Zhang, X. Wu, Y. Xu, Q. Guo, M. A. Subramanian, W. F. Stickle, T. Wu, J. Lu and X. Ji, *Adv. Energy Mater.*, 2020, **10**, 2000968.
13. Y. Xu, X. Wu, H. Jiang, L. Tang, K. Y. Koga, C. Fang, J. Lu and X. Ji, *Angew. Chem. Int. Ed.*, 2020, **59**, 22007-22011.
